# GLP‐1 Receptor Agonism Exacerbates Activity‐Based Anorexia in Mice

**DOI:** 10.1002/eat.70169

**Published:** 2026-07-08

**Authors:** Amit Thakar, Travis E. Brown, Shane T. Hentges

**Affiliations:** ^1^ Department of Integrative Physiology and Neuroscience Washington State University Pullman Washington USA

**Keywords:** activity‐based anorexia, food intake, GLP‐1 receptor agonist, GLP‐1 receptor antagonist, mice, motivated behavior, restricted feeding, weight loss, wheel‐running

## Abstract

**Objective:**

Glucagon‐like peptide‐1 receptor (GLP‐1R) agonists reduce food intake and body weight, suggesting they should be avoided in anorexia nervosa. However, preclinical evidence that these drugs reduce activity and reward‐seeking behavior raises the possibility that they might attenuate the compulsive‐like hyperactivity central to ABA pathology and slow weight loss. Here, we employed a widely used rodent model of AN (activity‐based anorexia, ABA) to determine whether a GLP‐1R agonist would worsen or rather lessen weight loss and hyperactivity.

**Method:**

Female mice that had continuous access to a running wheel and time‐restricted food availability received systemic administration of a GLP‐1R agonist, a GLP‐1R antagonist, or vehicle once daily before food presentation (*N* = 10/group).

**Results:**

GLP‐1R agonist treatment exacerbated ABA‐associated weight loss, confirming the concern that appetite suppression would worsen outcomes and failing to support the possibility that reduced compulsive‐like activity might provide benefit. This effect was driven by a further suppression of food intake, while wheel running remained unchanged. Pharmacological blockade of GLP‐1R signaling had no detectable effect on any parameter monitored relative to vehicle controls.

**Discussion:**

These findings provide the first experimental evidence that GLP‐1R agonism worsens outcomes in an animal model of anorexia nervosa. The lack of effect of GLP‐1R antagonism suggests endogenous GLP‐1 signaling does not substantially contribute to ABA pathology. Together, these data raise caution regarding the use of GLP‐1R agonists in individuals vulnerable to restrictive eating as they are unlikely to reduce the motivation for excessive activity and can further reduce food intake.

## Summary

- Medications known as GLP‐1 receptor agonists are widely used to treat obesity and diabetes because they reduce appetite and body weight, but very little is known about how they may affect people vulnerable to restrictive eating disorders such as anorexia nervosa. Their well‐known appetite‐suppressing effects suggest these drugs could worsen anorexia nervosa by further reducing food intake; however, because GLP‐1R agonists have also been shown to attenuate compulsive reward‐seeking behaviors, a counterintuitive hypothesis is that they might instead reduce the excessive, compulsive‐like exercise that drives anorexia pathology and thereby lessen its severity.
- Using a well‐established mouse model of anorexia nervosa, this study found that a GLP‐1 receptor agonist worsened unhealthy weight loss by further reducing food intake without reducing excessive exercise‐like activity.
- Blocking GLP‐1 receptors did not alter food intake, activity, or weight loss, suggesting that natural GLP‐1 signaling may not strongly drive anorexia‐like behaviors in this model.
- As the use of GLP‐1 medications continues to rapidly expand, this research raises important concerns about their potential risks in individuals who are underweight, prone to restrictive eating, or recovering from anorexia nervosa.

## 1 Introduction

Glucagon‐like peptide‐1 receptor agonists (GLP‐1RAs) robustly suppress food intake, reduce body weight, and improve glycemic control, leading to their widespread clinical use for obesity and type 2 diabetes (Drucker 2018). Beyond metabolic effects, early preclinical studies demonstrated that GLP‐1RAs reduce basal (Erreger et al. 2012) and stimulant‐induced (Erreger et al. 2012; Sorensen et al. 2015) locomotor activity, and more recent work indicates that these agents can attenuate motivation for both food and drug rewards in rodents and humans (Badulescu et al. 2024; Eren‐Yazicioglu et al. 2020; Hayes and Schmidt 2016). Together, these findings have prompted growing interest in GLP‐1R signaling as a regulator of motivated behavior and as a potential therapeutic target for disorders characterized by compulsive reward seeking, including substance use disorders (Yammine et al. 2025; Shen et al. 2024; Martinelli et al. 2024). The parallels between anorexia nervosa (AN) and substance use disorders in terms of continued behaviors despite negative consequences and shared neurobiological underpinnings in reward and motivation circuitry (Kaye et al. 2013), make it plausible that GLP‐1R agonists could reduce aberrant behavior in AN. Whether the ability of GLP‐1R agonists to reduce motivated behavior could override the ability to decrease food intake in AN remains an important question and is the focus of this brief preclinical study.

Despite their profound effects on feeding and motivation, the relevance of GLP‐1RAs to eating disorders marked by restrictive intake has received little attention. Evidence from binge‐eating disorder suggests that GLP‐1RAs can reduce binge frequency, consistent with their capacity to suppress excessive consummatory behavior (Radkhah et al. 2025; Aoun et al. 2024). However, whether these drugs exert beneficial, neutral, or harmful effects in AN—a disorder defined by extreme food restriction, weight loss, and often excessive physical activity—remains largely unknown.

This gap is particularly concerning given the rapid expansion in availability and off‐label use of GLP‐1RAs for weight loss. These trends raise the possibility that GLP‐1RAs could be misused by individuals who are underweight, vulnerable to restrictive eating, or recovering from AN. To date, published evidence addressing this risk is limited to isolated clinical reports, including a case describing worsening of atypical AN following GLP‐1RA misuse (Liekens et al. 2025). Notably, no controlled preclinical studies have systematically examined the effects of GLP‐1R modulation in an established animal model of AN.

Activity‐based anorexia (ABA) is the most widely used rodent model of AN and recapitulates key features of the disorder by combining time‐restricted feeding with continuous access to a running wheel (Spadini et al. 2021; Schalla and Stengel 2019). This paradigm induces severe weight loss driven by both reduced food intake and paradoxical hyperactivity. Based on the known anorectic and locomotor‐suppressing effects of GLP‐1RAs in people (Drucker 2018; Jensen and Torekov 2021), two competing predictions emerge. On one hand, GLP‐1R agonism might be expected to exacerbate ABA‐associated weight loss by further suppressing intake. On the other, if GLP‐1RAs reduce activity or general motivation, they could potentially attenuate ABA severity by limiting excessive wheel running. Disentangling these possibilities is essential for understanding how GLP‐1R signaling interacts with pathological motivational states.

In the present study, we used the ABA model in mice to examine how pharmacological modulation of GLP‐1R signaling influences food intake, wheel running, and body weight loss. We tested both a GLP‐1R agonist and a GLP‐1R antagonist under a moderately restrictive feeding condition. While GLP‐1R antagonists have been reported to increase food intake and body weight in other contexts (Meeran et al. 1999; Turton et al. 1996), their effects during ABA have not been previously assessed. Our findings demonstrate that GLP‐1R agonism markedly exacerbates ABA‐associated weight loss by selectively suppressing food intake without reducing hyperactivity, whereas GLP‐1R antagonism has no detectable impact on ABA outcomes. These results reveal a striking context‐dependent dissociation between the effects of GLP‐1R signaling on feeding and activity and raise important concerns regarding the use of GLP‐1RAs in individuals vulnerable to restrictive eating.

## 2 Methods

All procedures were approved by the Washington State University Animal Care and Use Committee (Protocol #7099) and conducted in accordance with the National Institutes of Health Guide for the Care and Use of Laboratory Animals. Female C57BL/6J mice were obtained from The Jackson Laboratory (Bar Harbor, ME) and housed under a phase‐shifted 12‐h light/dark cycle (lights off at 10:00) with controlled temperature (20°C–22°C) and constant humidity. Water was provided *ad libitum* throughout the study, and standard rodent chow (Product #5001; Animal Specialties, Quakertown, PA) was available *ad libitum* until the start of the activity‐based anorexia (ABA) paradigm when food was present for the first 4 h of the dark period each day.

Upon arrival from the supplier, mice were acclimated to the facility and the phase‐shifted light/dark cycle for 10 days in group housing. After acclimation, they were single‐housed in cages equipped with running wheels (Catalog #0297; Columbus Instruments, Columbus, OH) for 3 days to acclimate to single housing and the running wheel. This was followed by a 5‐day period where baseline food intake, running wheel activity, and bodyweight were recorded daily. During the 5‐day baseline period, all animals had *ad libitum* access to food and water. On each of the last 3 days of the baseline period, all mice received a saline injection 0.5 h prior to lights‐out to habituate them to the injection procedure.

At the end of the baseline period, 10 mice were assigned to each condition: Saline, GLP‐1R agonist (Exendin_4_), or GLP‐1R antagonist (Exendin_9–39_), and were matched for body weight and running‐wheel activity. Exendin_4_ (HY‐13443) and Exendin_9–39_ (HY‐P0264) were purchased from Hello Bio (Princeton, NJ). During the ABA phase, animals received an IP injection of saline, GLP‐1R agonist (50 μg/kg), or GLP‐1 antagonist (50 μg/kg) 0.5 h before food was presented at the start of the dark cycle. Food remained available for 4 h. Body weight and food intake continued to be recorded daily. Bodyweight was taken each day just prior to the injection. Running‐wheel activity was recorded continuously in 15‐min bins using Multi‐Device Interface Software (Columbus Instruments). Animals were removed from the study once they reached 80% of baseline body weight or upon completion of Day 10 of the ABA paradigm.

All data were analyzed using Prism (GraphPad Software Inc., San Diego, CA). Statistical tests used for individual analyses are described in the Results section. Data are presented as mean ± SEM, and statistical significance was defined as *p* ≤ 0.05.

## 3 Results

In the present study we used a relatively mild ABA paradigm where the mice were allowed access to normal chow for 4 h each day at the start of the dark cycle (Figure 1A). This paradigm recapitulates key features of the 2 h food access ABA protocol that we have used in published studies (Bailey et al. 2026; Christensen et al. 2025; Daimon and Hentges 2021, 2022) including decreased food intake (Figure 1B), the development of wheel‐running in the hours before dark/food presentation (food‐anticipatory activity; Figure 1C), and rapid weight loss (Figure 1D). However, mice with 4 h daily food access increased their food intake as the paradigm was continued (Figure 1B); ABA Day 0 average food intake 1.76 ± 0.09 g, ABA Day 10 average food intake 3.53 ± 0.16 g (t(9.642), *p* < 0.0001, df = 18) and maintained their bodyweight above threshold for removal (80% of baseline; Figure 1D dotted line) through the full 10 days of testing whereas mice maintained with 2 h feeding maintain minimal food intake and reach threshold for removal within 2–4 days (Bailey et al. 2026; Christensen et al. 2025; Daimon and Hentges 2021, 2022). This longer food access paradigm was utilized to probe the effect of GLP‐1R manipulation to allow for better detection of treatment‐induced exacerbation of time to removal threshold should that occur while still allowing assessment of altered running and food intake.

**FIGURE 1:**
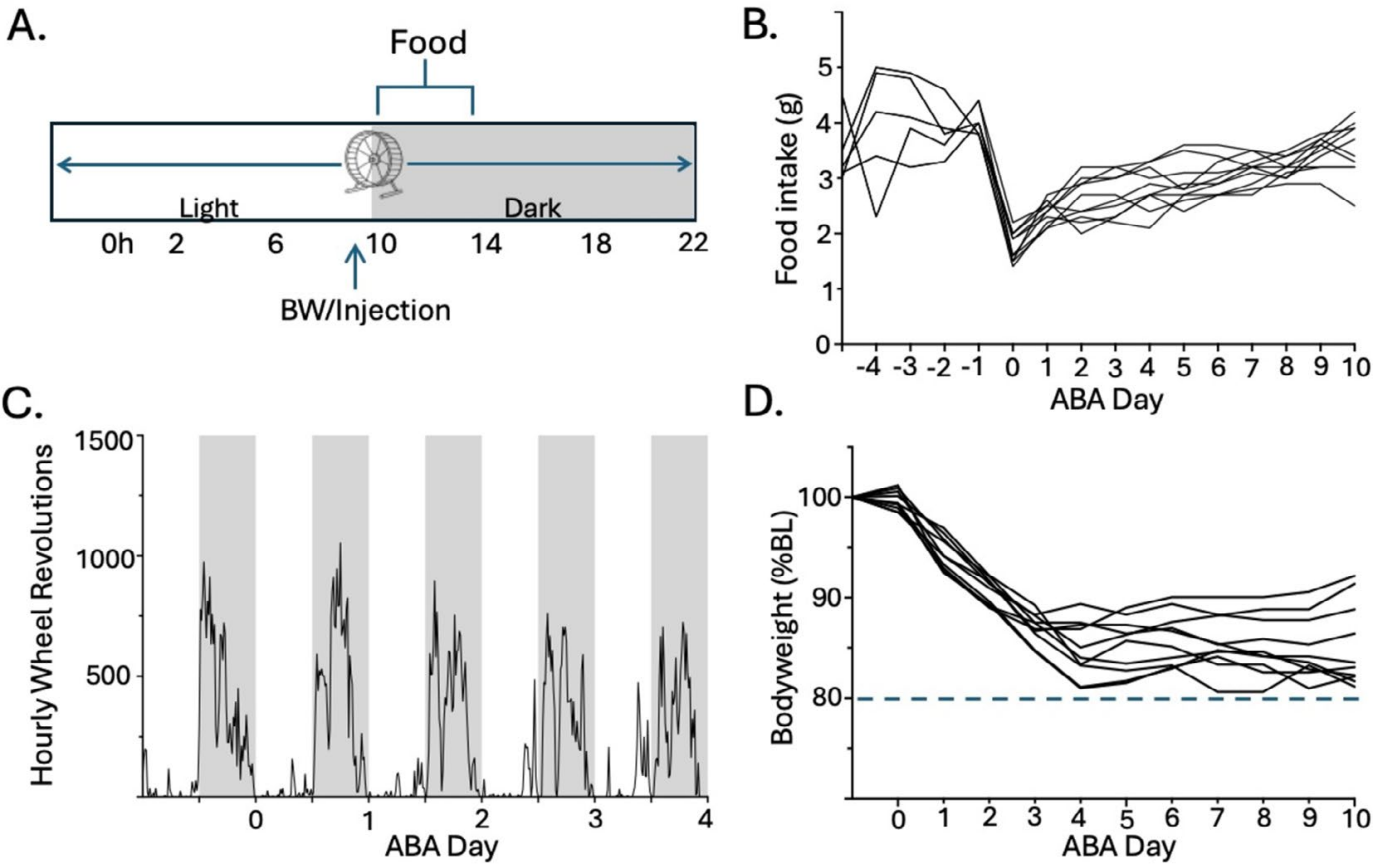
Mild ABA paradigm. Food presented for 4 h at the start of the dark (starting at ABA Day 0) coupled with an available running wheel (A) leads to an acute reduction in food intake that lessens over time (B). While modest, light‐period wheel running that develops over days of ABA (C). Bodyweight declines rapidly early in this ABA paradigm compared to baseline (BL) but stabilizes above the removal threshold (dashed line) around Day 4 (D). The blue arrow in (A) indicates when bodyweight (BW) was recorded each day and when the injection was given. Control mice are presented here and all received saline. *N* = 10, except panel C where the average of 1 experiment with 5 mice is shown. The gray bars in (C) indicate the dark periods of each day.

Using the 4 h food presentation paradigm, we found that mice that received the GLP‐1R agonist exendin_4_ (50 μg/kg, 1 × daily just prior to the dark period, Figure 2A, blue bars) showed reduced food intake compared to saline treated controls (Figure 2A, black bars) on all days of ABA (Days 0–4), whereas the antagonist exendin_9‐39_ with the same dosing parameters did not alter food intake compared to saline (Figure 2A, green bars) as detected with a mixed effects model (significant effect of day (*p* < 0.001; F_3,75_ = 46.0), treatment (*p* < 0.001; F_2,27_ = 21.3), and day x treatment (*p* = 0.009, F_8,103_ = 2.73)). Daily wheel running was not statistically different between groups (Figure 2B; treatment *p* = 0.395; F_2,27_ = 0.96 for Days 0–3 of ABA), although both the agonist (blue circles) and antagonist (green squares) groups displayed somewhat higher wheel running than the group receiving saline (open black circles). While total daily wheel‐running was not statistically different between treatment groups, running was more apparent prior to food presentation in both the agonist and antagonist groups (Figure 2C, running in non‐gray/light times) than in saline‐treated mice (Figure 1C, very modest daytime running). Only mice treated with the GLP‐1R agonist lost 20% or more of their baseline bodyweight during the restricted feeding days (Figure 2D, blue line).

**FIGURE 2:**
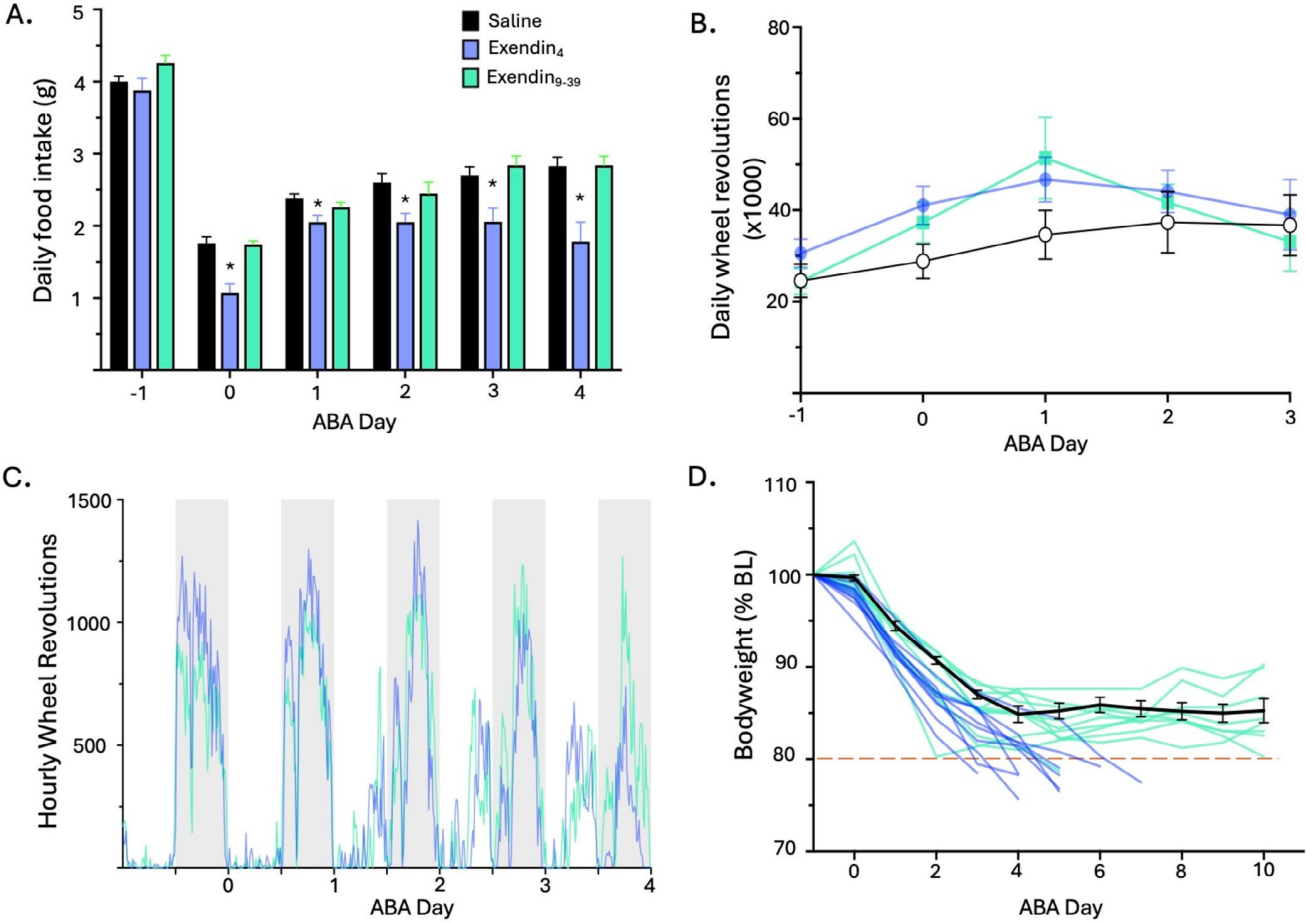
GLP‐1R manipulation and physiologic response to the ABA paradigm. Mice receiving the GLP‐1R agonist Exendin4 (A, blue bars) on Days 0–4 showed decreased food intake each day compared to saline treated mice (A, black bars), whereas the antagonist Exendin_9‐39_ did not alter food intake in the paradigm (A, green bars). Neither the agonist nor antagonist altered daily wheel running (B) and both groups showed similar day/night running patterns (C; gray areas indicate dark periods). Mice receiving the agonist (blue line) lost significantly more bodyweight and had to be removed from the experiment as they reached 80% of baseline weight (D, dashed line). * = *p* < 0.05 using Tukey's multiple comparison post hoc test. All groups started with *N* = 10, although panel C shows data from the average of 1 experiment with 5 mice.

## 4 Discussion

The present study demonstrates that GLP‐1R agonism exacerbates weight loss during ABA in mice by suppressing food intake without reducing excessive wheel running. In contrast, GLP‐1R antagonism did not significantly alter food intake, wheel running, or body weight loss. These findings provide the first direct experimental evidence that GLP‐1R activation worsens outcomes in a preclinical model of anorexia‐like behavior.

Although the appetite‐suppressing properties of GLP‐1R agonists raised concern that these drugs might worsen ABA, prior reports that they can reduce locomotor activity and motivated behaviors led us to also consider the counterintuitive possibility that GLP‐1R activation might attenuate ABA severity by limiting pathological running (Erreger et al. 2012; Sorensen et al. 2015; Badulescu et al. 2024; Eren‐Yazicioglu et al. 2020; Hayes and Schmidt 2016). We found that a dose of Exendin_4_ previously shown to reduce basal locomotion in rodents (Erreger et al. 2012) selectively reduced food intake while leaving wheel‐running behavior largely intact. This dissociation suggests that the mechanisms driving hyperactivity in ABA may differ from the motivational processes typically suppressed by GLP‐1R signaling. Together with previous studies showing that agents like leptin and opioid receptor antagonists can reduce the hyperactivity in ABA without increasing food intake (Daimon and Hentges 2021; Hillebrand et al. 2005) it appears that each aspect of ABA might be driven independently.

The lack of detectable effect of GLP‐1R antagonism was somewhat unexpected as its ability to increase food intake has been shown in some studies (Turton et al. 1996; Williams et al. 2009). However, the ABA model represents an extreme motivational and metabolic condition in which powerful competing neurobiological drives may dominate behavioral output. Under these conditions, endogenous GLP‐1 signaling may contribute minimally to the suppression of feeding relative to other mechanisms engaged by restricted feeding, stress, heightened arousal, or excessive exercise. Alternatively, it is possible that endogenous GLP‐1 tone during ABA is already low or functionally overridden, limiting the efficacy of receptor blockade, or that the selected dose was insufficient to override any basal GLP‐1 tone.

The findings here may have translational relevance given the rapidly increasing clinical and off‐label use of GLP‐1R agonists for weight loss. Although evidence regarding their effects in anorexia nervosa remains limited, our data support concerns that GLP‐1R agonists could exacerbate restrictive eating and pathological weight loss in vulnerable individuals (Bartel et al. 2024; Leziak et al. 2025).

Several limitations of this brief study are acknowledged. Only female mice and a single dosing regimen were studied, and ABA models capture only selected features of AN. The dose of exendin_4_ was chosen based on several detailed studies in rats and mice with dose responses reported (Erreger et al. 2012; Sorensen et al. 2015; Egecioglu, Engel, and Jerlhag 2013; Mack et al. 2006; Egecioglu, Steensland, et al. 2013). The selected dose of agonist reflects a relatively high dose that has been shown to reduce general locomotion in previous work (Erreger et al. 2012). Observing no inhibition of wheel‐running and significantly blunted food intake at the chosen dose, we felt that testing additional doses was unwarranted as higher doses would likely cause greater weight loss and lower doses would be unlikely to reduce running as they do not affect basal running. Future studies could extend the antagonist dose further as the dose used here is modest. However, there is little evidence that GLP‐1R antagonists increase basal food intake, and we saw no suggestion of efficacy in the ABA paradigm. An additional limitation is the inclusion of only one agonist. Exendin_4_ (exenatide) is an early GLP‐1R agonist that has been heavily used in preclinical studies but is less commonly used in people given that more potent later generation agonists exist. However, exendin4 likely has more central actions than other agonists (Salameh et al. 2020), suggesting that others may be even less likely to reduce parameters of ABA or AN.

In summary, GLP‐1R agonism worsened ABA by selectively suppressing food intake without reducing pathological wheel running, whereas GLP‐1R antagonism had no detectable effect. These findings reveal a context‐dependent dissociation between the effects of GLP‐1R signaling on feeding and hyperactivity and suggest that GLP‐1R agonists may exacerbate restrictive eating pathology under vulnerable conditions. As the clinical use of GLP‐1R agonists continues to expand, understanding their effects in populations susceptible to restrictive eating disorders will be increasingly important.

## Author Contributions

**Shane T. Hentges:** writing – original draft, visualization, conceptualization, formal analysis, methodology, funding acquisition. **Amit Thakar:** conceptualization, writing – review and editing, investigation, visualization, methodology. **Travis E. Brown:** conceptualization, writing – review and editing, funding acquisition.

## Lived Experience Involvement Statement

No specific efforts were undertaken to involve persons with lived experience in the study design or execution, or in the preparation of this manuscript.

## Funding

This work was funded by WSU startup funds and an intramural college of veterinary medicine research award to T.E.B. and S.T.H.

## Ethics Statement

All experiments were approved by the Institutional Animal Care and Use Committees at Washington State University in accordance with the National Institute of Health's Guide for the Care and Use of Laboratory Animals.

## Conflicts of Interest

The authors declare no conflicts of interest.

## Data Availability

The data that support the findings of this study are available from the corresponding author upon reasonable request.
